# Impact of Pre-Treatment Strategies on Enzymatic Hydrolysis of Alternative Protein Sources: Case Study for Black Soldier Fly Larvae

**DOI:** 10.3390/molecules31101770

**Published:** 2026-05-21

**Authors:** Sandra Borges, Tânia C. F. Ribas, André Almeida, Manuela Pintado

**Affiliations:** 1 CBQF—Centro de Biotecnologia e Química Fina—Laboratório Associado, Escola Superior de Biotecnologia, Universidade Católica Portuguesa, Rua Diogo Botelho 1327, 4169-005 Porto, Portugal; tpedro@ucp.pt (T.C.F.R.); mpintado@ucp.pt (M.P.); 2 SEBOL—Comércio e Indústria do Sebo, S.A., 2660-119 Loures, Portugal; andre.almeida@etsa.pt; 3 I.T.S.—Indústria Transformadora de Subprodutos, S.A., 2100-406 Coruche, Portugal

**Keywords:** pre-treatment, sustainable protein sources, enzymatic hydrolysis, alcalase, bioactivity

## Abstract

The rising global demand for protein-rich food has intensified interest in alternative and sustainable protein sources. Insects, particularly black soldier fly (BSF) larvae, represent promising substrates due to their high nutritional content and potential for valorization into functional ingredients. This study investigated the impact of pre-hydrolysis treatments on the efficiency of enzymatic hydrolysis using alcalase to enhance protein solubilization and bioactive peptide production. Pre-treatments included organic acids (propionic and acetic acid) and a pressure-thermal method. Results indicated that BSF larvae responded differently to the evaluated pre-treatment strategies. Notably, the pressure-thermal treatment combined with enzymatic hydrolysis increased soluble protein content by approximately 30% and antioxidant activity by approximately 20%, suggesting enhanced release of bioactive peptides. Although organic acid treatments increased protein solubility, they did not improve the degree of hydrolysis or antioxidant activity. These findings highlight the potential of pressure-thermal pre-treatment to improve the efficiency of protein extraction from insect biomass and support the integration of such approaches into food bioprocessing strategies aimed at developing novel, high-value protein ingredients.

## 1. Introduction

The rapid growth of the global population has intensified the challenges faced by the food and agriculture sectors, particularly in ensuring a sustainable and resilient food supply chain. Over the past decade, shifting consumption patterns and increasing demand for protein have placed unprecedented pressure on conventional agricultural systems, which are already constrained by limited arable land, environmental degradation, and the escalating impacts of climate change [1]. In response, there is a growing emphasis on identifying innovative and sustainable protein alternatives within the framework of a circular economy. Among the emerging alternatives, insect-derived hydrolysates have garnered significant attention due to their high nutritional value and reduced environmental footprint [2]. In particular, black soldier fly (BSF) larvae have emerged as a promising substrate, owing to their high feed conversion efficiency, short life cycle, and rich composition of proteins, fats, and essential minerals [3]. The substantial protein content of BSF makes it a compelling candidate for valorization through enzymatic hydrolysis to yield high-value bioactive compounds [4].

Recent studies have increasingly explored the enzymatic hydrolysis of BSF proteins to improve their nutritional, functional, and bioactive properties. Different enzymes, including alcalase [5], papain [6], bromelain [7], and flavourzyme [5], have been evaluated for their ability to generate BSF hydrolysates with distinct peptide profiles and bioactivities, demonstrating the relevance of hydrolysis conditions on the characteristics of the final products. The BSF hydrolysates have demonstrated diverse bioactivities (such as antioxidant [8], antihypertensive [4], and anti-inflammatory [9] properties) and possess physicochemical characteristics suitable for food and feed applications [10].

In recent years, research has increasingly focused on optimizing protein hydrolysis processes to maximize the functional and nutritional value of alternative protein sources [11]. One promising approach involves the application of pre-treatment strategies prior to enzymatic hydrolysis to enhance hydrolysis efficiency and improve the quality of the resulting hydrolysates [12]. In particular, non-thermal processing technologies such as ultrasound (US), high-pressure processing (HPP), and pulsed electric field (PEF) have gained considerable attention as pre-treatment methods due to their ability to improve protein extraction and the bioactivity of the generated peptides [12]. Therefore, this study investigated the effects of pressure-thermal and organic acid (propionic or acetic acid) pre-treatments on the enzymatic hydrolysis of BSF larvae. These strategies aim to disrupt structural barriers and facilitate enzyme accessibility, thereby improving protein yield and the bioactive properties of the hydrolysates [13,14]. To our knowledge, the application of such pre-treatments in the context of insect protein hydrolysis remains underexplored, with limited studies available to date [15].

## 2. Materials and Methods

### 2.1. Materials and Reagents

The ETSA Group (Loures, Portugal) supplied the dehydrated and milled BSF larvae. Unless otherwise specified, all reagents were obtained from Sigma-Aldrich (St. Louis, MO, USA).

### 2.2. Composition Analysis

The composition of BSF larvae was analyzed following the methods outlined by the Association of Official Analytical Chemists [16]. Protein content was measured using the Kjeldahl method, moisture content was determined by drying the samples at 105 °C for 24 h, and ash content was quantified by heating the samples at 550 °C for 5 h.

### 2.3. Pre-Treatments and Enzymatic Hydrolysis

Three groups of pre-treatments were applied to the BSF larvae, namely (i) pressure-thermal pre-treatment using high pressure and temperature (ii) acetic acid pre-treatment, and (iii) propionic acid pre-treatment. For the pressure-thermal pre-treatment, 10% (*w*/*v*) raw material was suspended in 0.1 M potassium phosphate buffer (pH 8) (i.e., 2 g of BSF larvae per 20 mL of buffer solution) and then subjected to high pressure (15 psi) and temperature (121 °C) for 15 min. For the acidic pre-treatments, BSF larvae suspensions were prepared at a final concentration of 10% (*w*/*v*) by mixing 2 g of BSF larvae with either 2% (*v*/*v*) acetic acid or 2% (*v*/*v*) propionic acid, followed by incubation at 50 °C for 1 h. After the acidic pre-treatments, the pH was adjusted to 8 using 6 M NaOH, in accordance with the manufacturer’s recommendations for alcalase (Alcalase^®^ 2.4 L, Novozymes, Bagsværd, Denmark), a serine endoprotease derived from *Bacillus licheniformis*, for subsequent enzymatic hydrolysis.

Enzymatic hydrolysis was then performed using a method adapted from Borges et al. [17]. The hydrolysis was performed using 1.0% (E/S, *v*/*w*, corresponding to 20 µL of enzyme per 2 g of substrate) alcalase for 4 h. Hydrolysis experiments were carried out in an orbital shaker (Thermo Scientific MaxQ 6000, Thermo Fisher Scientific, Waltham, MA, USA) at 125 rpm and 50 °C. At the end of the process, samples were heated at 100 °C for 15 min to inactivate the enzyme. The samples were then rapidly cooled, centrifuged at 5000 rpm for 30 min, and the supernatant was collected and stored at −20 °C for subsequent analyses.

Control assays included: (i) BSF larvae subjected only to different pre-treatment conditions without subsequent enzymatic hydrolysis and (ii) enzymatic hydrolysis performed without any pre-treatment of BSF larvae. All control conditions were prepared using the same substrate concentration and processing conditions described above. Each assay was performed in duplicate.

### 2.4. Characterization of Protein Hydrolysates

#### 2.4.1. Total Soluble Protein Content

Protein concentration was determined using the Pierce BCA Protein Assay Kit (Thermo Scientific, Waltham, MA, USA), with bovine serum albumin as the standard. The results were reported in mg/mL of hydrolysate.

#### 2.4.2. Degree of Hydrolysis

Samples for degree of hydrolysis (DH) determination were collected at different stages of the process to distinguish the effect of pre-treatments and enzymatic hydrolysis. Samples were withdrawn (i) before pre-treatment (raw material), (ii) immediately after each pre-treatment, and (iii) at the end of the enzymatic hydrolysis.

The DH was determined by measuring the concentration of free amino groups through their reaction with 2,4,6-trinitrobenzenesulfonic acid (TNBS), following the method described by Sousa et al. [18]. The reaction mixture consisted of 50 µL of the sample, 125 µL of 200 mM sodium phosphate buffer (pH 8.2), and 50 µL of 0.025% TNBS solution in deionized water, prepared in a 96-well microplate (Sarstedt, Nümbrecht, Germany). The mixture was incubated at 50 °C for 1 h, and absorbance was measured at 340 nm. A standard curve was generated using L-leucine at 50 to 250 µM. All conditions were analyzed in duplicate.

DH was calculated using the formula:

DH (%) = (*L*_t_ − *L*_0_)/(*L*_max_ − *L*_0_) ×100

where *L*_t_ is the amino groups released at hydrolysis time, *L*_0_ is the free amino groups at time zero (blank), and *L*_max_ is the total of amino groups in the raw material. BSF larvae were acid hydrolyzed with 6 M HCl at 105 °C for 24 h to obtain the *L*_max_. Before evaluating the amino groups, the acid-hydrolyzed sample was filtered, and the supernatant was neutralized with 6 M NaOH.

The DH values corresponding to pre-treatments and enzymatic hydrolysis were calculated by considering the increase in free amino groups after each pre-treatment and the final hydrolysis time (4 h) relative to the initial raw material, using the same *L*_0_ and *L*_max_ definitions described above.

#### 2.4.3. Antioxidant Activity

The antioxidant activity of samples was assessed using the 2,2′-azinobis (3-ethylbenzothiazoline-6-sulfonic acid) (ABTS) radical scavenging assay, following the protocol described by Castro et al. [19]. An ABTS^+^ stock solution was prepared by mixing equal volumes (1:1, *v*/*v*) of 7 mM ABTS and 2.44 mM potassium persulfate, both prepared in ultrapure water, and incubated in the dark for 16 h at room temperature. For the analysis, the ABTS^+^ working solution was diluted with ultrapure water to achieve an absorbance of 0.70 ± 0.02 at 734 nm. A standard curve was generated using Trolox at 25 to 175 µM. Briefly, in a 96-well microplate, 20 µL of the sample, Trolox standard, or water (control) was added in triplicate, followed by 180 µL of the ABTS^+^ working solution. After a 6 min incubation at 30 °C, absorbance was measured at 734 nm using a microplate reader. Results were expressed as Trolox equivalents per liter of hydrolysate (µmol eq. Trolox/L). Each sample was analyzed in triplicate.

#### 2.4.4. Molecular Weight Profile

High-Performance Size Exclusion Chromatography (HPSEC) was conducted to analyze the peptide profile of samples, following the methodology described by Fernandez Cunha et al. [20]. The analysis employed an Agilent AdvanceBio SEC column (Agilent Technologies, London, UK) with a particle size of 2.7 μm, pore size of 130 Å, and dimensions of 7.8 mm inner diameter × 300 mm length. The column was eluted under isocratic conditions with a phosphate buffer (0.15 M NaH_2_PO_4_, pH 7) at a flow rate of 1 mL/min.

Filtered samples (10 μL) were injected, and detection was performed using a Waters 2690 system equipped with a photodiode array (PDA) detector operating in the 190–600 nm range. Data analysis was conducted with Empower 3 software. Molecular weights (MW) of chromatographic peaks were determined by referencing a calibration curve prepared with protein standards: Ovalbumin (44,300 Da), Myoglobin (17,600 Da), Cytochrome C (12,327 Da), Aprotinin (6511 Da), Neurotensin (1672 Da), Angiotensin-II (1040 Da), Tyr-Phe dipeptide (328.4 Da), and L-tryptophan (204 Da).

HPSEC analysis was performed for the hydrolysate obtained by enzymatic hydrolysis without pre-treatment and for the hydrolysate corresponding to the condition that showed the most pronounced effects among the evaluated treatments, in order to compare the MW distribution profiles between untreated and pre-treated samples.

### 2.5. Statistical Analysis

Data were analyzed using a *t*-test with a significance level of 0.05, performed with the Statistical Package for the Social Sciences (SPSS) software, version 21 (Armonk, NY, USA). Results were reported as the mean values of at least two replicates.

## 3. Results and Discussion

### 3.1. Composition Analysis of BSF Larvae

The analysis of the composition of dried BSF larvae powder used in the hydrolysis process is crucial, as it offers valuable insights into the raw material that can significantly influence the characteristics and quality of the resulting hydrolysate. The proximate composition of the dried BSF larvae utilized in this study is presented in Table 1. The protein content was determined to be 35.3 g per 100 g of substrate, aligning closely with values reported in previous research on dried BSF larvae composition [3]. This consistency supports the reliability of the material used and provides a foundation for comparing the outcomes of the hydrolysis process across different studies.

### 3.2. Characterization of BSF Larvae Protein Hydrolysates

**Figure 1:**
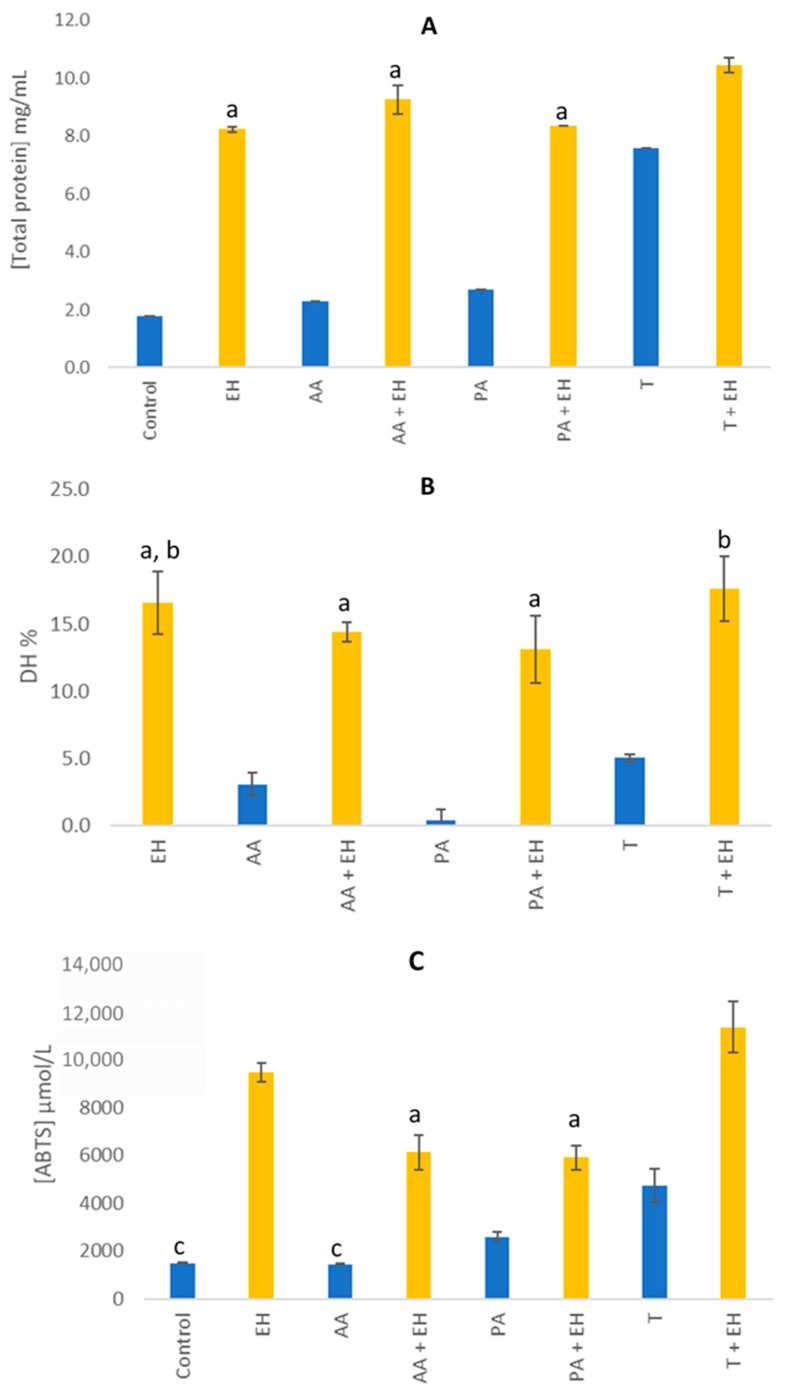
Characterization of BSF larvae hydrolysates produced with different pre-treatments. (**A**) Total soluble protein content by BCA; (**B**) degree of hydrolysis by TNBS; (**C**) antioxidant activity by ABTS. a, b, c Same lowercase letters indicate no significant difference (*p* > 0.05). Control, no pre-treatment and no enzymatic hydrolysis; EH, enzymatic hydrolysis; AA, acetic acid pre-treatment; AA + EH, acetic acid pre-treatment followed by enzymatic hydrolysis; PA, propionic acid; PA + EH, propionic acid pre-treatment followed by enzymatic hydrolysis; T, pressure-thermal pre-treatment; T + EH, pressure-thermal pre-treatment followed by enzymatic hydrolysis.

Figure 1 illustrates the effects of different pre-treatments, including acetic acid, propionic acid, and high pressure and temperature, on the enzymatic hydrolysis of BSF larvae, focusing on soluble protein content (Figure 1A), degree of hydrolysis (Figure 1B), and antioxidant activity (Figure 1C).

The application of acetic acid, propionic acid, or the pressure-thermal pre-treatment without enzyme significantly increased soluble protein levels of the raw material compared with the control (*p* < 0.05) (Figure 1A), indicating enhanced protein extraction promoted by the pre-treatments. Among the evaluated pre-treatments, the pressure-thermal condition resulted in the highest increase in soluble protein content. When combined with enzymatic hydrolysis, a significant improvement was observed only for the pressure and temperature plus enzyme condition compared with enzymatic hydrolysis alone (*p* < 0.05), resulting in an increase of approximately 30% in soluble protein. The enhanced protein solubility observed after the pressure-thermal treatment may be associated with the disruption of cellular and organelle structures, which can increase protein accessibility for extraction and enzymatic hydrolysis. In addition, the pre-treatment may have promoted structural modifications in the proteins, facilitating enzyme access to cleavage sites [21,22]. Li et al. [21] described similar effects, where heat pre-treatment of clam (*Aloididae aloidi*) significantly enhanced the soluble peptide content of hydrolysates. It is important to note that the effects on protein solubility can vary depending on the insect species; for instance, pressure pre-treatment enhanced the solubility of mealworm hydrolysate compared to control hydrolysate, while it reduced the solubility of cricket hydrolysate [15].

Regarding the DH (Figure 1B), the combination of enzymatic treatment with the acidic or the pressure-thermal pre-treatment did not result in substantial increases in DH compared with enzymatic hydrolysis alone. It has been reported that DH stabilizes once the substrate is fully hydrolysed, with no further increase beyond this point [23,24]. On the other hand, a decrease in proteolysis was observed when acidic pre-treatments were used, compared to the pressure-thermal treatment (*p* < 0.05). These results indicated that acid pre-treatment was not favorable for hydrolysis, potentially due to structural modifications in proteins induced by acetic and propionic acids. These alterations likely reduced enzyme-substrate interactions, thereby decreasing DH [25].

Regarding the antioxidant activity (Figure 1C), the pre-treatment strategies influenced the bioactivity of hydrolysed BSF proteins. Notably, the combination of high temperature and pressure with enzymatic hydrolysis resulted in the highest antioxidant activity, having an increase of ca. 20%. The increase in antioxidant activity aligns with the higher soluble protein content observed in Figure 1A, reinforcing the idea that protein hydrolysis liberates peptides with enhanced bioactive properties. This could indicate that groups capable of scavenging the ABTS radical were released, and a higher content was available in the BSF hydrolysate. Enhanced antioxidant activity was also observed in deboned chicken residual hydrolysates pre-treated by high-pressure (200 MPa) [25] and in pumpkin seed hydrolysates pre-treated by sonication [26]. On the other hand, a decrease in antioxidant activity was noted when acidic pre-treatments were used, which could be due to protein aggregation through hydrophobic interactions [25].

Among all tested conditions, the combination of pressure-thermal pre-treatment with enzymatic hydrolysis yielded the highest values across all parameters, indicating its superior efficacy in enhancing protein solubility, hydrolysis efficiency and bioactivity of BSF-derived peptides.

**Figure 2:**
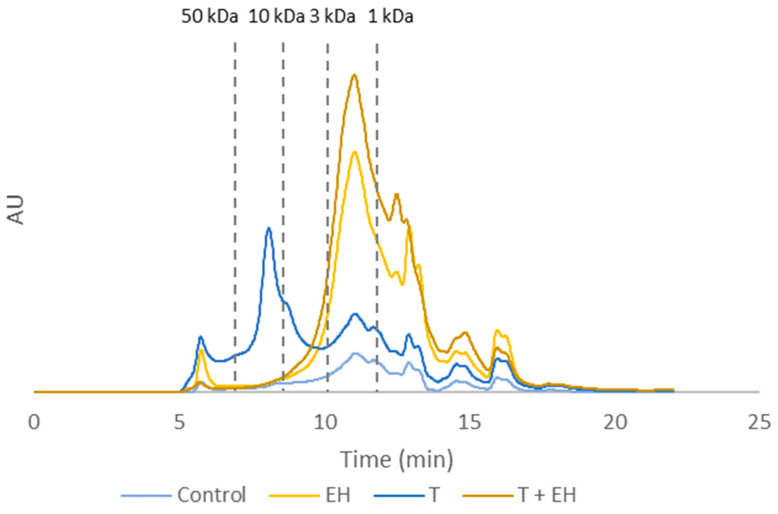
MW distribution of BSF hydrolysates obtained under different treatment conditions. Control, no pre-treatment and no enzymatic hydrolysis; EH, enzymatic hydrolysis; T, pressure-thermal pre-treatment; T + EH, pressure-thermal pre-treatment followed by enzymatic hydrolysis.

The MW profile of BSF hydrolysates revealed that enzymatic hydrolysis promoted the formation of low MW peptides, particularly in the region below 10 kDa (Figure 2). The combination of the pressure-thermal pre-treatment with enzymatic hydrolysis showed a higher relative intensity of peptides in lower MW, especially below 3 kDa, compared with the other evaluated conditions (Figure 2). Similar effects have been reported for sonication pre-treatment, which generated a high proportion of peptides below 1 kDa [25].

This pre-treatment, followed by alcalase hydrolysis, could be beneficial for producing bioactive peptides or enhancing protein digestibility. As previously mentioned, an increase in antioxidant activity and soluble protein content of BSF hydrolysate was observed. Wang et al. [27] similarly reported that ultrasound treatment of soybean protein reduced β-sheet components and exposed hydrophobic sites, enhancing enzyme accessibility and leading to increased radical scavenging activity. These effects were attributed to the greater exposure of functional groups and a higher proportion of low MW peptides with strong antioxidant properties following the alcalase hydrolysis of pre-treated soybean protein.

These findings support the potential of combining pressure-thermal pre-treatment with enzymatic hydrolysis to improve the production of low MW BSF peptides with potential bioactive properties.

## 4. Conclusions

The exploration of new protein sources derived from edible insects has gained increasing attention in the past decade due to their potential economic and environmental benefits. This study investigated the use of pre-treatment of BSF larvae before the hydrolysis procedure with alcalase. The treatment processes evaluated were the use of a pressure-thermal procedure and the use of an organic acid (acetic and propionic acids) before enzymatic hydrolysis to enhance hydrolysis efficiency. A comparison between the different approaches (enzymatic hydrolysis with no pre-treatment and with the different pre-treatments) was assessed in terms of the total soluble protein content of the hydrolysate, degree of hydrolysis, and antioxidant capacity. The results indicated that for BSF larvae, the pressure-thermal treatment led to an increase in total soluble protein content (≈30%) and antioxidant activity (≈20%). This suggests that pressure-thermal pre-treatment could be a promising strategy to improve hydrolysis efficiency for the production of a high-value protein ingredient, promoting a more sustainable use of resources.

## Figures and Tables

**Table 1:** Proximate composition of dried BSF larvae.

	BSF Larvae
Protein (%)	35.3 ± 0.4
Ash (%)	5.2 ± 0.2
Moisture (%)	7.5 ± 0.1
Lipids (%)	34.1 ± 2.5
Carbohydrate (%) *	≈18%

* Calculated as the difference in all composition components.

## Data Availability

The original contributions presented in this study are included in the article. Further inquiries can be directed to the corresponding author.
